# Neutralization by Insulin of the Hypertensive Effect of Dermcidin Isoform 2: An Environmentally Induced Diabetogenic and Hypertensive Protein

**DOI:** 10.1155/2014/412815

**Published:** 2014-02-04

**Authors:** Rajeshwary Ghosh, Sarbashri Bank, Rabindra Bhattacharya, Nighat N. Khan, A. Kumar Sinha

**Affiliations:** ^1^Sinha Institute of Medical Science and Technology, 288 Kendua Main Road, Garia 700 084, India; ^2^Gandhi Memorial Hospital, Kalyani, West Bengal 741245, India; ^3^RR&D Center of Excellence, James J. Peters VA Medical Center, 130 W. Kingsbridge Road, Bronx, NY 10468, USA

## Abstract

The effect of dermcidin isoform 2 (dermcidin), an environmentally induced stress protein, was investigated on the genesis of diabetes mellitus and hypertension, the two major atherosclerotic risk factors. The role of dermcidin as an atherosclerotic risk factor related to the impaired systemic insulin level was investigated. Dermcidin was prepared by electrophoresis using plasma from the subjects with acute ischemic heart disease. Injection of 0.2 *μ*M dermcidin in mice increased the blood glucose level from 98 ± 2.45 mg/dL to 350  ± 10.2 mg/dL which was normalized by the oral administration of acetyl salicylic acid (aspirin) after 24 h. Hypertensive subjects with systolic and diastolic blood pressure of 165 mm and 95 mm of Hg, respectively, had plasma dermcidin level of 95 nM. Ingestion of acetyl salicylic acid (aspirin) (150 mg/70 kg body weight) decreased the systolic and diastolic pressures to 125 mm and 80 mm of Hg, respectively, with decrease of dermcidin level to 15 nM. Incubation of kidney cortex cells with 0.2 *μ*M dermcidin-inhibited synthesis of (r)-cortexin, an antihypertensive protein, and the basal (r)-cortexin level was reduced from 33 nM to 15 nM. Addition of 25 *μ*units of insulin/mL was found to reverse the inhibition of cortexin synthesis. The effect of dermcidin as a diabetogenic and a hypertensive agent could be controlled either by aspirin or by insulin.

## 1. Introduction

The etiology of essential hypertension (EH), a major form of hypertension in man (>90%), remains obscure [1]. More importantly, the nature of the regulatory component involved in the control of the disease remains obscure [1]. Despite the fact that hypertension is considered to be a genetic disease, the discovery of a gene that regulates the blood pressure in man is yet to be identified [2]. Although extensive studies from all over the world have shown the presence of various chromosome regions, no specific genes responsible for EH have yet been identified [3]. On the other hand, several environmental stress factors are also reported to be involved in the genesis of EH in man [4, 5]. In this context, an environmentally induced stress protein identified to be dermcidin isoform 2 (dermcidin) has been reported [6] which was found to be both a diabetogenic and a hypertensive agent posing an important threat for the development of atherosclerosis [7]. Although various risk factors including hypoxia and tobacco smoke may induce the synthesis of dermcidin, no systemic regulatory control is yet known for dermcidin synthesis. However, in this context we have reported that acetyl salicylic acid (aspirin) and nitric oxide can counteract the effects of the stress-induced protein [6, 8]. Although reports are available suggesting that hyperglycemia can cause hypertension [9, 10], no mechanism on the hypertensive effect of hyperglycemia, in the context of insulin resistance, is currently available. In this context, we have reported the isolation of an antiessential hypertensive protein from the kidney cortex cells of goat, nominally called renal (r)-cortexin which can control the elevated blood pressures through the stimulation of endothelial synthesis of NO, a global antihypertensive agent [11]. It was further found that (r)-cortexin was present in the plasma of all mammals tested including humans [11]. The plasma level of (r)-cortexin in hypertensive people was found to be reduced to 0 pmol/mL that contrasted the optimal range of >200 pmol/mL in the normal counterparts [11]. The antihypertensive protein not only was found to control *l*-epinephrine induced elevated blood pressures (both systolic and diastolic pressures) in the animal model but also was found to play a critically important role in the control of essential hypertension in humans [11].

In an effort to find out the role of insulin on the synthesis of (r)-cortexin, investigations were carried out particularly in the context of dermcidin synthesis that was reported to be both a diabetogenic and hypertensive protein [7]. We also report herein the neutralization of the dermcidin-induced atherosclerotic risk by insulin and by aspirin via the synthesis of NO through the control of both hyperglycemia and hypertension.

Although the development of naturally occurring environmental stresses in animals under laboratory conditions is difficult to achieve, investigations were also carried out to determine the effect of various stresses on animals by exposing these animals to pure nicotine, hypoxia, and aqueous extract of tobacco leaves.

The aggregation of platelets is a critically important physiologic event in the life saving blood coagulation process [12]. In contrast, the formation of thrombus (microaggregate of platelets embedded in fibrin mass) due to excessive platelet aggregation, at the site of atherosclerotic plaque rupture or fissuring on the coronary artery, has been reported to result in acute ischemic heart disease (AIHD) [13]. As diabetes and hypertension are the two major risk factors for AIHD, the possible role of dermcidin as an aggregating agent was studied. On the other hand, since insulin has been reported to be a global antithrombotic agent [14], the role of insulin in the inhibition of dermcidin-induced platelet aggregation was also investigated.

## 2. Materials and Methods

### 2.1. Ethics Statement

The protocols used in this study were approved by the Internal Review Board, Sinha Institute of Medical Science and Technology, Kolkata. These studies used the blood samples from normal volunteers and from patients suffering from AIHD or those with EH. Appropriate consent form was signed by the participants before they were requested to donate blood.

Required approval was also obtained for the use of animals in the study by the Internal Review Board for Animal Care, Sinha Institute of Medical Science and Technology, Kolkata, consisting of a special committee for animal care and its use that oversaw the welfare, care, and nutritional requirements of all the animals used in the study. The committee consisted of a permanent certified veterinarian whose duty was to ensure that all the animals were free from any diseases as stipulated by the Animal Right Group. All animal-related experiments were strictly performed in the presence of a member of the Animal Right Group and under the supervision of the veterinarian and special care was taken to ensure that no animals were unnecessarily harmed or were subjected to pain during the study. After the termination of the study, the animals were sacrificed by euthanasia in a carbon dioxide chamber.

White albino healthy mice (20–25 gm each), Swiss strain, irrespective of gender, were used for the study [15]. These inbred animals were fed with standard laboratory chow and sterilized water was given *ad libitum. *The animals were kept under 12 h cycles of light and dark at 23°C.

### 2.2. Chemicals

Goat anti-rabbit immunoglobulin G-alkaline phosphatase, nicotine, was purchased from Sigma Aldrich. Enzyme-linked immunosorbent assay (ELISA) MaxiSorp plates were from Nunc, Roskilde, Denmark. Aspirin was obtained from Medica Zydus Healthcare.

### 2.3. Selection of Hypertensive Persons

The details of the selection of hypertensive patients have been described recently [7]. The selected patients (*n* = 40; M = 25, F = 15) who participated in the study were “outdoor patients” who had clinically undefined malaise. Only those patients who at presentation were unaware of their hypertensive condition and, as such, those who never received any treatment for the elevated blood pressures were included in the study. Subjects with systolic blood pressure (SBP) ≥140 mm of Hg and diastolic blood pressure (DBP) ≥90 mm of Hg were considered to be hypertensive [1] and were selected for the study. A sphygmomanometer was used for measuring the BP.

### 2.4. Selection of the Patients Suffering from Acute Ischemic Heart Disease

Details of the selection of the patients suffering from AIHD have also been reported before [6]. Briefly, these patients (*n* = 10; M = 7, F = 3) had characteristic chest pain for more than 30 mins and had characteristic ST elevation in EKG. The occurrence of AIHD was confirmed by the determination of the plasma CKMB isoenzyme within 6 h of the chest pain before the initiation of any therapy.

### 2.5. Collection of Blood

Blood samples (20–25 mL) were collected in plastic vials by venipuncture from the participants in sodium citrate as an anticoagulant [16] [9 vol blood : 1 vol of the anticoagulant (0.013 M final concentration)] using 19 gauge siliconized needles before the initiation of any therapy for the condition. Cell free plasma (CFP) was prepared by centrifuging the blood sample at 30,000 g for 30 min at 0°C.

### 2.6. Preparation of Dermcidin

Dermcidin used in the study was prepared from the plasma of AIHD patients by sequential polyacrylamide gel electrophoresis first in the presence and then in the absence of sodium dodecyl sulphate to retain its biological activity as described before [6].

### 2.7. Development of ELISA for Insulin and Dermcidin

To determine the plasma insulin and dermcidin levels, ELISA was performed as described before [6] using the polyclonal antibodies raised against insulin and purified dermcidin, respectively [6].

### 2.8. The Effects of Hypoxia, Aqueous Tobacco Leaf Extract, and Pure Nicotine on the Plasma Dermcidin Levels in Mice

Group A mice (*n* = 5) were raised in our animal care facility as described above and were subjected to hypoxic condition equivalent to 1/2 atmospheric pressure using vaccum pump for 60 min at 22°C. Blood samples (0.5 mL) were collected from the tail vein as described before [17].

Group B mice (*n* = 5) were fed with 0.1 mL of the tobacco leaf extract prepared by steeping 1.0 g of tobacco leaves in 1.0 mL of 0.9% NaCl overnight. The leaves free aqueous portion (0.1 mL) was then administered orally to the mice and 0.5 mL of blood samples was collected from the tail vein at different times [17].

Group C mice (*n* = 5) were injected with 50 pmol of nicotine/25 g body weight in 0.9% NaCl. Blood samples were collected as described above at different times.

Dermcidin levels in the blood samples (appropriately diluted) were determined by ELISA as described before [6].

### 2.9. Immunoblot Analysis of Dermcidin in the Plasma of Hypertensive Patients

The presence of dermcidin in the plasma of hypertensive subjects treated with and without 150 mg of aspirin was identified by immunoblot technique [18]. Dermcidin was identified by using anti-dermcidin antibody as described elsewhere [8]. The intensity of the dermcidin bands was determined by using ImageJ software [8].

### 2.10. Preparation of Aspirin Solution

Fresh acetyl salicylic acid solution was prepared just before use by dissolving the compound in 0.1 M NaHCO_3_ and immediately neutralizing the solution to pH 7.0 at 0°C which was discarded after use [19].

### 2.11. Determination of (r)-Cortexin Synthesis in the Kidney Cortex Cells by *In Vitro* Translation of mRNA

Kidney cortex cells homogenate was prepared as described before [11]. The pelleted fraction was incubated in the presence or absence of different concentrations of dermcidin and/or insulin. The (r)-cortexin synthesis was determined by *in vitro* translation of the extracted (r)-cortexin mRNA from the cells as described previously by using plant leaf ribosomal particles in the presence of 1.0 mM ATP and 1.0 *μ*M of all 20 amino acids [11].

### 2.12. Determination of Blood Glucose Level

The blood glucose level was determined by using a glucometer (Behringer).

### 2.13. Aggregation of Platelets

The aggregation of platelets was carried out individually in 10 different platelet rich plasma (PRP) samples from 10 different normal subjects (F = 5; M = 5) by incubating each PRP sample with electrophoretically purified *≈*1.0 *μ*M dermcidin in an aggregometer as described [6, 19]. In parallel experiments, 100 *μ*units of insulin was added along with *≈*1.0 *μ*M dermcidin to the PRP samples to study the effect of insulin on the inhibition of platelet aggregation induced by dermcidin. The quantitation of percent aggregation was determined from the initial angle of the platelet aggregation profile.

### 2.14. Statistical Analysis

The results shown are mean ± standard deviation (SD). Each experiment was carried out at least 10 times using 5-6 different animals or blood samples from 40 hypertensive subjects and 10 normal volunteers each in triplicate. The significance of the results was analyzed by Student's paired *t*-test where difference in the mean values was considered significant with *P* < 0.05. Additionally, one-way ANOVA analysis was performed using Bonferroni's multiple comparison test and the Newman-Keuls multiple comparison test wherever applicable with a significance of *P* < 0.05 using GraphPad Prism. The coefficient of correlation (r) was determined by Pearson's test.

## 3. Results

### 3.1. The Effect of Some Well-Known Environmental Stresses on the Synthesis of Dermcidin in Mice

Although environmental stresses have been implicated in the development of various life-threatening conditions, including hypertension [4, 5], diabetes mellitus [20], and many other diseases, the identity of the mediators however for these conditions remains obscure [21, 22]. In 2011, we have reported, for the first time ever, the association of dermcidin with acute myocardial infarction and also reported that the systemic increase of nitric oxide decreased the plasma dermcidin level for a better prognostic outcome of the condition [6]. The association of dermcidin in the environmentally induced type 1B diabetes mellitus (T1BDM) and in atherosclerosis has also been reported recently [7, 23].

**Figure 1:**
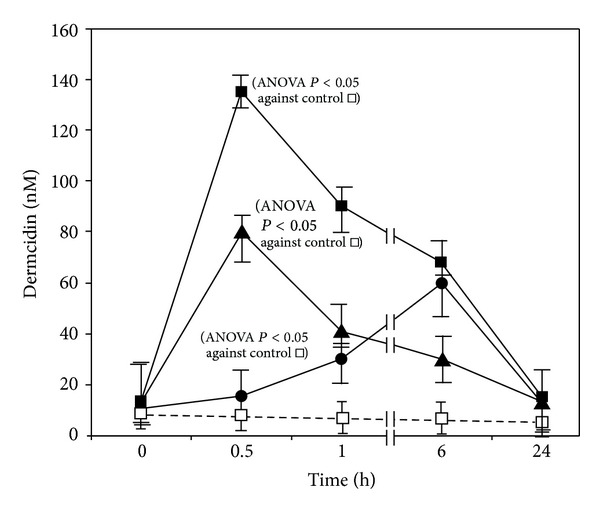
The effect of different stress factors on the systemic increase of dermcidin level. Three groups of mice (*n* = 5 in each group) were exposed to pure nicotine, tobacco leaves free aqueous extract, and hypoxia. The dermcidin level was measured in the blood of these animals as described in Section 2 by ELISA using anti-dermcidin antibody at different times as shown. Solid squares (■) represent the plasma level of dermcidin in mice injected with 50 pmol of nicotine/25 g body weight. Solid triangles (▲) represent the dermcidin level in mice administered with 0.1 mL of the tobacco leaves free aqueous extract. Solid circles (●) indicate the dermcidin level in mice exposed to hypoxia. Hollow squares (□) indicate the dermcidin level in mice not subjected to any stress factors. The results shown are mean ± SD of 10 different experiments using different animals, each in triplicate.

As mentioned elsewhere, it is very difficult to reproduce the naturally occurring environmental stresses in animals under laboratory conditions. However, the effects of several known environmental stresses like hypoxia, use of tobacco leaf, or pure nicotine in mice were investigated. The time course of the plasma dermcidin level in mice exposed to the stress factors is shown in Figure 1. The significance in the elevation of the dermcidin level in mice treated with either nicotine or tobacco leaf extract or hypoxia in comparison to the control group was analyzed by one-way ANOVA test using Newman-Keuls multiple comparison test which suggested that the increase in the dermcidin level was significant with *P* < 0.05 compared to the control experiment.

### 3.2. Effect of Injection of Dermcidin in the Circulation on the Development of Hyperglycemia due to Impaired Plasma Insulin Level in Animal Model

**Figure 2:**
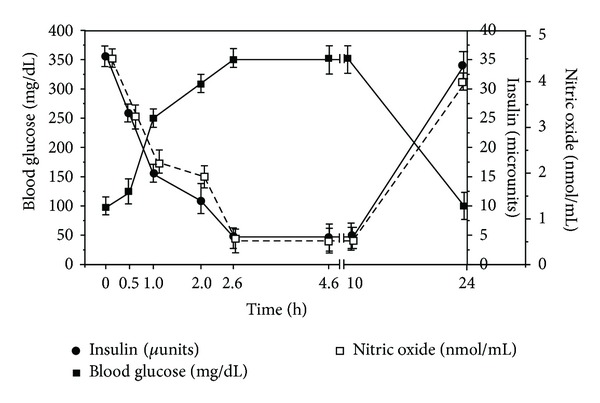
The *in vivo* effect of dermcidin on the blood glucose and plasma insulin levels in mice. Normal adult mice were injected with 0.2 *μ*M purified dermcidin in the tail vein and their blood glucose, plasma insulin, and NO levels were recorded at different times as shown. Solid squares (■) represent the blood glucose level in these animals at different times. Solid circles (●) demonstrate the insulin level at different times. Hollow squares (□) represent the corresponding NO level at different times as indicated. The figure represents data expressed in mean ± SD of 10 different experiments using 6 different animals, each in triplicate.

As demonstrated in Figure 1, several environmental stress factors are responsible for the increase of dermcidin level in mice. In this context, we have recently reported the occurrence of elevated dermcidin level in the plasma of T1BDM patients by >120 nM reported to be induced by environmental stresses [23]. To study the *in vivo* effect of dermcidin on the blood glucose and plasma insulin level in mice, 0.2 *μ*M dermcidin was injected in the tail vein of the test animals and the blood glucose and insulin levels were determined at different time intervals (Figure 2). The blood glucose level which was 98 ± 2.45 mg/dL before the injection of dermcidin was found to be elevated to 350 ± 10.2 mg/dL at 160 min after the injection of the stress-induced protein with concomitant decrease of the plasma insulin level from 35.56 ± 2.42 *μ*units/dL to 4.56 ± 0.018 *μ*units/dL after the injection. Further studies demonstrated that the blood glucose level of 350 ± 10.2 mg/dL as well as the insulin level of 4.56 ± 0.018 *μ*units/dL remained nearly unchanged for the next 120 mins (Figure 2). However, after 24 h of dermcidin injection, both blood glucose levels and insulin levels were found to return to their normal ranges. When the level of dermcidin was measured in the same animals, it was found that the plasma dermcidin level which was 9.98 ± 2.2 nM before the injection was increased to 65.5 ± 2.8 nM after 160 mins of dermcidin injection. However after 24 h, the dermcidin level was found to be reduced to near normal level of 11.5 ± 2.34 nM. On the other hand, in the control experiment where the animals were not subjected to dermcidin injection, the blood glucose and the plasma insulin level remained at normal ranges of 96 ± 2.5 mg/dL and 37.2 ± 3.1 *μ*units/dL, respectively, with the dermcidin level at 10.69 ± 2.57 nM at different times. The results of the different groups (both experimental and control groups) were subjected to one-way ANOVA test which showed that the means as well as the variances were significantly different with *P* < 0.05. The Newman-Keuls multiple comparison test further suggested that the difference between the means of the different groups was significant with *P* < 0.05. A *t*-test analysis was also performed to confirm the significance of the results obtained and it was found that the two-tailed *P* value was significant with alpha = 0.05.

In a separate experiment, the effect of aspirin, a reported antagonist of dermcidin *in vivo* [6, 8], was studied by orally administering 0.125 mg aspirin/25 g mice together with 0.2 *μ*M dermcidin injection. The corresponding levels of insulin, glucose, and NO were found to be within the normal ranges of 30 ± 3.3 *μ*units/dL, 96 ± 5.2 mg/dL, and 4.1 ± 0.24 nmol/mL, respectively. The dermcidin level was also found to be in the normal value of 11.1 ± 2.0 nM. One-way ANOVA test as well as paired *t*-test indicated that the difference between the means of the results of the groups treated with dermcidin in the presence and absence of aspirin was significant (*P* < 0.05).

As a corollary, it could be inferred from these results that the impaired systemic insulin synthesis, as induced by dermcidin, might actually result in the increase of the plasma levels of the stress-induced protein.

### 3.3. The Role of Dermcidin-Induced Genesis of Hypertension in Humans and the Reversal of Its Effect by Acetyl Salicylic Acid

We have reported before that the injection of 1.0 nmol dermcidin/kg body weight in rabbit increased the SBP and DBP by 76% and 46%, respectively, within 60 mins and the dermcidin-induced elevated blood pressures returned to normal ranges during the next 4 h [7].

**Figure 3:**
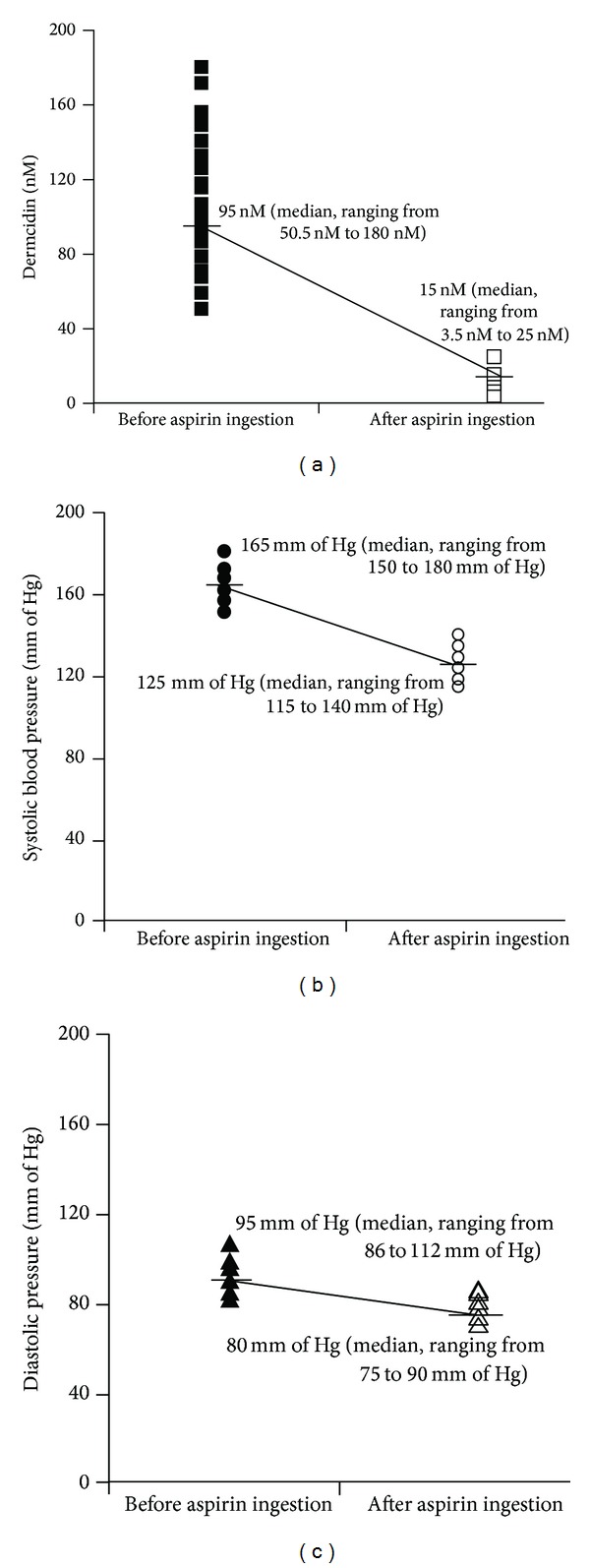
The effect of the ingestion of aspirin on the dermcidin level and systolic and diastolic blood pressure levels in humans. Aspirin at a bolus dose of 150 mg/70 kg body weight was ingested orally by hypertensive subjects as described above. The plasma dermcidin level and blood pressure level (both systolic and diastolic) were determined in these patients before and after the ingestion of the compound. Panel (a) represents the dermcidin level in the hypertensive subjects. Solid squares (■) represent the dermcidin levels before the ingestion of aspirin. Hollow squares (□) indicate the dermcidin levels after the ingestion of aspirin by the hypertensive subjects. Panel (b) represents the systolic blood pressure in the hypertensive subjects. Solid circles (●) demonstrate the systolic blood pressure level before the ingestion of aspirin. Hollow circles (○) show the systolic blood pressure after the ingestion of the compound. Panel (c) demonstrates the diastolic blood pressure in the subjects suffering from hypertension. Solid triangles (▲) represent the level of diastolic blood pressure before the oral ingestion of aspirin. Hollow triangles (∆) indicate the diastolic blood pressure levels after the ingestion of aspirin. The figure is a typical representative of 10 different experiments using the blood samples of 40 hypertensive volunteers (m = 25, f = 15), 35–45 years, each experiment in triplicate.

As it was not possible to determine the effect of dermcidin on the elevation of blood pressures in humans, when the plasma dermcidin levels in the hypertensive persons (*n* = 40; m = 25, f = 15) with elevated SBP level of 165 mm of Hg (median) and DBP level of 95 mm of Hg (median) were determined, it was found that the plasma dermcidin level was 95 nM (median, ranging from 50.5 nM to 180 nM) (Figure 3(a)).

A significant and positive correlation between the plasma dermcidin and blood pressure levels of the hypertensive subjects was observed when compared to the age and gender matched normal volunteers (*r* = +0.988).

**Figure 4:**
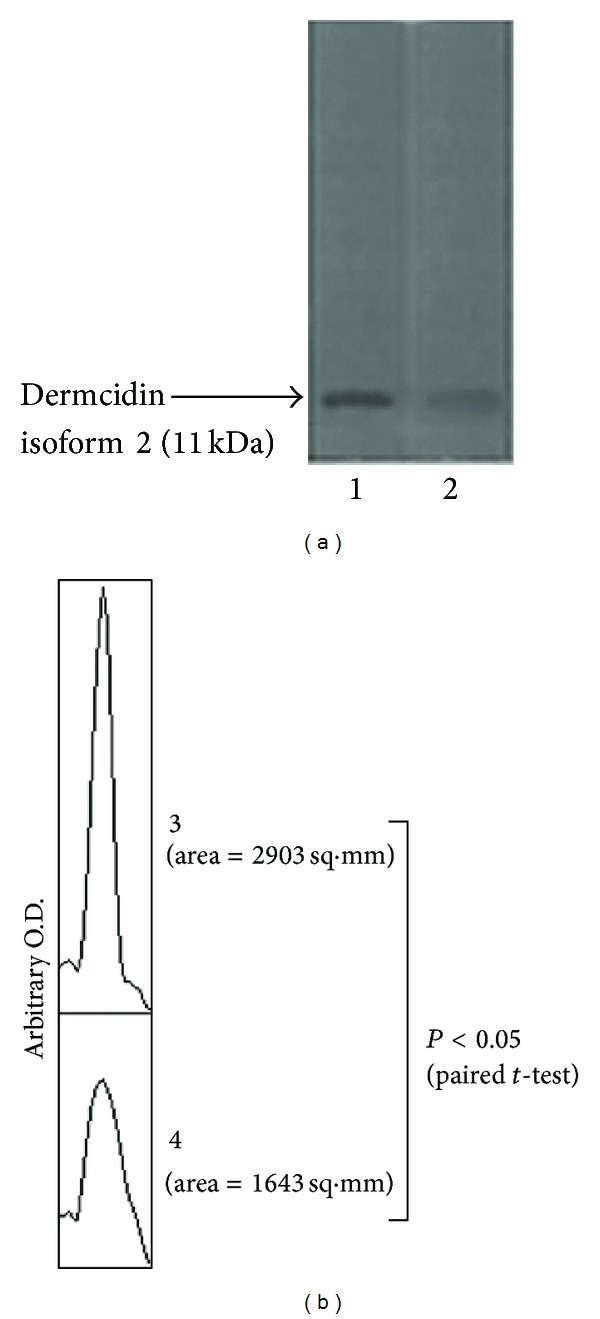
Immunoblot analysis of the plasma dermcidin level in hypertensive subjects before and after the ingestion of aspirin. The plasma from hypertensive persons before and after the ingestion (150 mg/70 kg) of aspirin was subjected to immunoblot analysis using dermcidin antibody as described in Section 2. The intensities of the bands obtained were determined using Image J software. Panel (a): 1 represents dermcidin band before the ingestion of aspirin, 2 represents dermcidin band after the oral ingestion of the compound. Panel (b): 3 represents corresponding profile plot of the intensity of the dermcidin band in the hypertensive patients before the ingestion of aspirin, 4 represents the corresponding intensity of the dermcidin band after the ingestion of the compound in the same patients. The figure is a typical representative of at least 10 different experiments using 10 different samples from 10 different volunteers (male = 5, female = 5).

As aspirin has been reported before to counteract the effect of dermcidin through the generation of systemic NO when ingested orally [6], the effect of the oral ingestion of aspirin on the plasma dermcidin and blood pressure levels was investigated. It was found that aspirin when taken orally at doses of 150 mg/70 kg body weight reduced the dermcidin level from 95 nM to 15 nM (Figure 3(a)) within 3 h of ingestion of the compound with a simultaneous decrease in the SBP (from 165 mm of Hg to 125 mm of Hg) (Figure 3(b)) as well as DBP (from 95 mm of Hg to 80 mm of Hg) (Figure 3(c)). Further investigations were made to determine the effect of the oral ingestion of aspirin on the plasma dermcidin level by subjecting the cell free plasma obtained from the hypertensive persons, before and after the ingestion of aspirin, to immunoblot analysis by using dermcidin antibody. As shown in Figure 4, the dermcidin band in the gel was quantitated by arbitrary OD, where the intensity of the band in the case of hypertensive plasma after the ingestion of aspirin was markedly less compared to that before the ingestion of the compound. To determine the significance of the results obtained, a paired *t*-test was done to find out the significance of the difference in the intensities of the dermcidin bands before and after the oral ingestion of aspirin by the hypertensive subjects. When the areas of the bands (Figure 4) were subjected to paired *t*-test, the mean value was found to be significantly different (*P* < 0.05) with a two-tailed *P* value of 0.0007.

### 3.4. The Role of Dermcidin on the Synthesis of (r)-Cortexin

**Figure 5:**
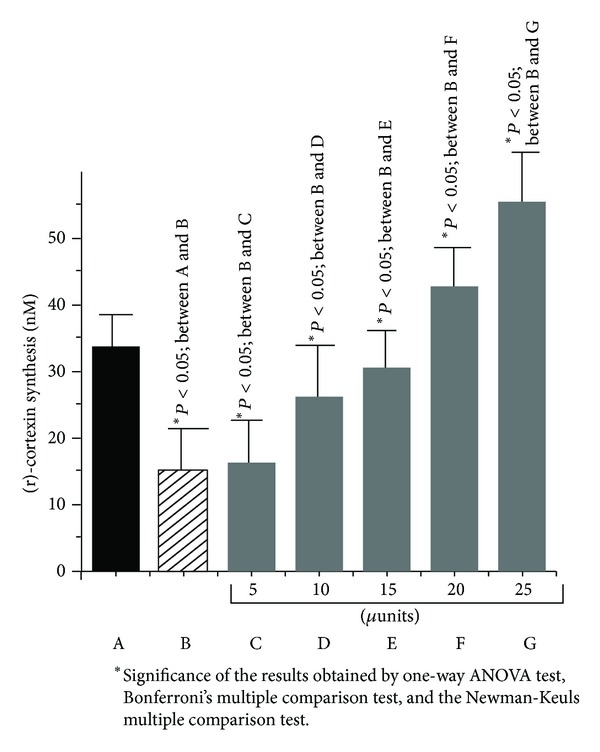
The opposing effect of dermcidin and insulin on the synthesis of (r)-cortexin. Typically kidney cortex cells homogenate in Kreb's buffer, pH 7.0, was prepared as described before [11]. The incubation of the suspension with 0.2 *μ*M dermcidin in the presence and absence of different amounts of insulin was carried out as described in Section 2. The *in vitro* translation of the (r)-cortexin mRNA was done as described in Section 2 and the (r)-cortexin synthesis was quantitated by ELISA. Solid bar represents the control experiment using only kidney cortex cells. The patterned bar represents incubation of kidney cortex cells treated with 0.2 *μ*M dermcidin. Gray bars indicate the incubation of kidney cortex cells treated with 0.2 *μ*M dermcidin in the presence of different amounts of insulin (*μ*units/mL). The significance of the results obtained between the different groups (A–G) has been shown in the figure. The results shown are mean ± SD of 10 different experiments using 5 different goat kidney cortex cells preparation, each in triplicate.

As dermcidin was reported to be a potent inhibitor of nitric oxide synthase [7] and NO was a stimulator of (r)-cortexin synthesis [11], the effect of dermcidin in the synthesis of (r)-cortexin, an antiessential hypertensive protein [11] in the goat kidney cortex cells, [11] was studied. In separate experiments, dermcidin (0.2 *μ*M) was incubated in the presence or absence of varying concentrations of insulin ranging from 5 to 25 *μ*units, as a stimulator of NO synthesis [24], with the kidney cortex cells preparation [11] or with 2.5 nmol/mL NO solution (as a positive control) instead of insulin itself at 37°C for different times. The (r)-cortexin synthesis was determined by the *in vitro *translation of (r)-cortexin mRNA as described in Section 2. It was found that the presence of 0.2 *μ*M dermcidin in the incubation system alone inhibited cortexin synthesis by *≈*50% after 30 mins of incubation. The (r)-cortexin synthesis which was 33.52 ± 0.025 nM in the absence of dermcidin was reduced to 15.32 ± 0.021 nM in the presence of 0.2 *μ*M stress-induced protein in the incubation mixture (Figure 5). On the other hand, the cortexin synthesis in the presence of 25 *μ*units of insulin in the assay mixture was found to increase by >3-folds to 55.5 ± 0.036 nM from 15.32 ± 0.021 nM after 30 mins of incubation even in the presence of 0.2 *μ*M dermcidin. Similar results were found in the case of cortex cells incubated with NO solution in the presence of dermcidin where the cortexin synthesis increased from basal 32.34 ± 0.022 nM to 53.91 ± 0.035 nM. A one-way ANOVA test was performed to determine the significance of the results and it was found that the means were significantly different with *P* < 0.05. Additionally both Bonferroni's multiple comparison test and the Newman-Keuls multiple comparison test suggested that the difference between the data was significant with *P* < 0.05 as indicated in Figure 5.

These results suggested that insulin, a potent stimulator of NO in the kidney cells, as well as an essential hormone for carbohydrate metabolism [25], was capable of synthesizing the anti-essential hypertensive protein ((r)-cortexin) by counteracting the inhibitory effect of the hypertensive protein (i.e., dermcidin).

When dermcidin synthesis was determined in the same reaction mixture described above, it was found that the synthesis of dermcidin which was 26.75 ± 0.25 nM in the absence of insulin was reduced to 6.89 ± 0.004 nM in the presence of 25 *μ*units of insulin (two-tailed paired *t*-test suggested that the means were significantly different with *P* < 0.05). These results demonstrated that the presence of insulin in the assay mixture was actually capable of inhibiting the synthesis of dermcidin in the cortex cells of the kidney with simultaneous increase of (r)-cortexin synthesis through (r)-cortexin mRNA synthesis and not merely due to the release of the preformed (r)-cortexin from the kidney cortex cells.

### 3.5. Inhibition by Insulin of the Platelet Aggregation Induced by Dermcidin

**Figure 6:**
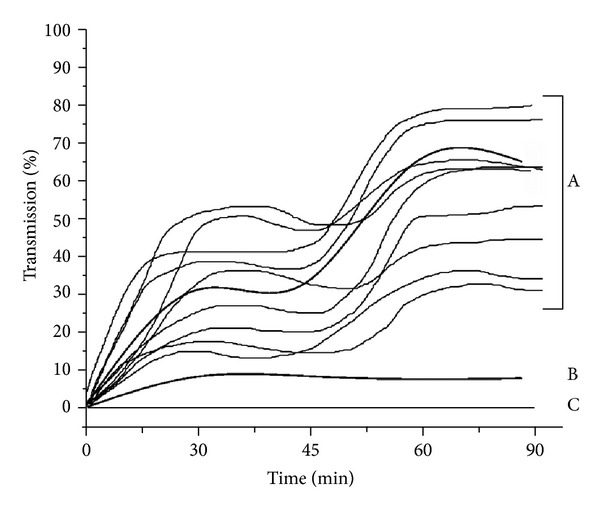
Effect of insulin on platelet aggregation in platelet rich plasma induced by electrophoretically pure dermcidin. Platelet rich plasma (PRP) was prepared as described in Section 2 and incubated with *≈*1.0 nM dermcidin for 90 min at 37°C. In a parallel experiment, PRP was incubated with the same amount of dermcidin in the presence of 100 *μ*units/mL insulin to determine the effect of the hormone on the dermcidin-induced platelet aggregation. The curves grouped under A represent the aggregation of the PRP incubated with *≈*1.0 nM dermcidin in 10 different PRP from 10 different normal volunteers. Curve B shows the inhibition of platelet aggregation incubated with *≈*1.0 nM dermcidin and 100 *μ*units insulin. Curve C represents the platelet aggregation of PRP in control experiment. The figure shown here is a typical representative of at least 10 different experiments using purified dermcidin in PRP from 10 different normal volunteers (m = 5; f = 5).

As the aggregation of platelets is a crucially important event in the development of AIHD due to the formation of thrombus at the site of atherosclerotic plaque rupture on the coronary artery [13], the contribution of dermcidin in the development of diabetes mellitus and hypertension should be taken into consideration in the context of the results described above. In this regard, the role of the stress-induced protein itself in the aggregation of platelets was carried out. Incubation of PRP with *≈*1.0 nM dermcidin was found to aggregate platelets (Figure 6). On the other hand, the incubation of PRP with 100 *μ*units of insulin in the presence of dermcidin nullified the proaggregatory effect of dermcidin completely.

## 4. Discussion

Environmentally induced stresses are reported to cause various life threatening conditions including hypertension and diabetes mellitus [4, 5, 20]. As these stresses may be created by diverse and sometimes unrecognized causes, the mechanism of the stress-induced diseases remains obscure [21, 22].

As described in Figure 1, different stress factors that include hypoxia, the use of tobacco leaf, and pure nicotine were found to be potent inducers of systemic dermcidin synthesis. Although no reports are available on the effects of environmental stresses on animals, particularly because the environmental stresses might not produce any effect in animals unlike in the case of human beings due to species differences, our results as described under Figure 1 suggested that these well-known stresses were capable of increasing dermcidin, which is reported to be a causative agent for both diabetes mellitus and hypertension [7]. We have also found that hypoxia, pure nicotine, and tobacco leaf extract were capable of expressing dermcidin gene within 30 mins of incubation in tissue culture of muscle cells, liver cells (primary culture), and endothelial cells of mice (unpublished) and also in the plasma of human volunteers exposed to hypoxia due to high altitude [26]. In other words, the effect of the stress inducers on dermcidin was the *de novo* synthesis of dermcidin on the expression of dermcidin gene as determined by the *in vitro* translation of dermcidin mRNA. We have reported before that dermcidin was capable of inducing T1BDM like condition due to the inhibition of glucose uptake both in the pancreatic islets of Langerhans and in the hepatocytes of the liver of adult mice [6]. As a consequence of the impaired glucose uptake in both kinds of cells, the sugar-induced synthesis of insulin was inhibited due to the impaired expression of both the proinsulin genes I and II in these cells [8, 27]. The human dermcidin was reported to be a potent inhibitor of all known forms of nitric oxide synthase (NOS) due to its ability to act as a competitive inhibitor of *l*-arginine, the only known substrate for all NOS [7]. The ability of dermcidin to inhibit insulin synthesis was found to be related to its effects on the synthesis of NO and it has been reported before that the synthesis of NO was critically important in the hepatic synthesis of insulin in the presence of glucose [8].

In this context, it should also be mentioned here that, despite repeated anecdotal claims that insulin resistance may lead to hypertension, no report is yet available on the mechanism of the development of hypertension in insulin resistance cases either in the case of type 2 diabetes mellitus or in the case of type 1 diabetes mellitus (T1DM). Indeed, the mechanism of the primary (essential) hypertension, the major form (<90%) of hypertension, until recently remained elusive. We, for the first time ever, reported that a kidney cortex derived antihypertensive protein ((r)-cortexin) was involved in the development of EH in man [11, 28]. We have also reported that dermcidin was not only a diabetogenic protein for T1BDM but also had a critically important role in the development of arterial hypertension through the inhibition of the synthesis of (r)-cortexin in the kidney cortex cells due to the inhibition of NO synthesis [29].

The results presented above described for the first time ever that the lack of systemic insulin synthesis might have led to the increased synthesis of dermcidin (Figure 5). It was also found that the use of physiologic range of insulin (25*μ*units/mL) was capable of increasing (r)-cortexin synthesis in the presence of dermcidin (Figure 5). These results, as such, demonstrated an etiological relation between the insulin resistance (i.e., the lack of systemic insulin) and the development of hypertension through the increased synthesis of dermcidin, a potent stimulator of arterial blood pressures (both systolic and diastolic) through the inhibition of (r)-cortexin synthesis in the kidney.

As dermcidin was capable of inhibiting insulin synthesis and as the lack of insulin synthesis was found to lead to increased dermcidin synthesis, a vicious circle would be implicated to worsen both T1BDM and hypertension due to the lack of systemic insulin synthesis. As atherosclerosis is reported to be the cause of increased incidences of cardiovascular and cerebrovascular conditions and as hypertension and diabetes mellitus are reported to be the two major risk factors for the development of atherosclerosis [30], the development of T1BDM might lead to increased atherosclerosis due to the lack of systemic insulin synthesis through the stimulation of the synthesis of dermcidin. Indeed, no mechanism of increased atherosclerosis in T1DM is currently available.

The chronic use of aspirin has been reported to reduce the occurrence of cardiovascular disease [31]. However, no mechanistic explanation of the aspirin effect on the reduction of atherosclerosis through the inhibition of cyclooxygenase that might lead to the control of either diabetes mellitus or hypertension is available. We have, on the other hand, reported that aspirin was an effective medication for the inhibition of dermcidin synthesis through the synthesis of NO [6, 8]. And, as such, the use of chronic aspirin in T1BDM might be helpful to prevent coronary artery disease through the inhibition of dermcidin synthesis as well as through increased systemic insulin synthesis due to the stimulation of nitric oxide synthase [6, 8]. The importance of aspirin-induced inhibition of cyclooxygenase in platelets in the reduction of cardiovascular diseases might have been overemphasized or speculative, in that the platelet aggregation in the absence of atherosclerosis in many cases is considered to be beneficial [7]. In this context it must be mentioned that dermcidin at nM ranges was found to induce platelet aggregation that could be inhibited by insulin (Figure 6). The results presented above taken together suggest the effects of the stress protein in the pathogenesis of various diseases including hypertension, hyperglycemia, and coronary artery disease, all of which are known to be involved in the development of AIHD which in turn could be controlled by insulin or aspirin through the synthesis of NO [6, 14].

As described above, the stimulated synthesis of dermcidin due to the lack of systemic insulin might play an important role in the development of atherosclerosis in T1BDM. In a preliminary study it was also found that, in type 1A diabetes mellitus, which is reported to be mediated by the immunologic assault of the pancreatic *β* cells [32], the plasma dermcidin level was found to be markedly increased (unpublished).

## 5. Conclusion

The systemic lack of insulin in T1DM where the plasma insulin level is known to vary from 0 to 10 *μ*units/dL might lead to the increased synthesis of dermcidin (Figure 5) which could be overcome in the presence of as low as 25 *μ*units of insulin/dL. And, as such, use of insulin itself might control essential hypertension. As insulin was found to inhibit synthesis of dermcidin, which is reported to induce platelet aggregation, the use of insulin might also prevent AIHD in T1DM through the inhibition of dermcidin synthesis.

Finally, it might be inferred that the stress-induced protein which has been reported to cause life-threatening diseases like hypertension, hyperglycemia, and AIHD could be controlled by insulin. Thus, insulin could have a global effect on the control of various diseases ranging from hypertension and diabetes mellitus to coronary artery disease.

## References

[1] Messerli FH, Williams B, Ritz E (2007). Essential hypertension. *The Lancet*.

[2] Harrap SB (2003). Where are all the blood-pressure genes?. *The Lancet*.

[3] Province MA, Kardia SLR, Ranade K (2003). A meta-analysis of genome-wide linkage scans for hypertension: the National Heart, Lung and Blood Institute Family Blood Pressure Program. *The American Journal of Hypertension*.

[4] Perry IJ, Whincup PH, Shaper AG (1994). Environmental factors in the development of essential hypertension. *British Medical Bulletin*.

[5] Pickering TG (1997). The effects of environmental and lifestyle factors on blood pressure and the intermediary role of the sympathetic nervous system. *Journal of Human Hypertension*.

[6] Ghosh R, Karmohapatra SK, Bhattacharyya M, Bhattacharya R, Bhattacharya G, Sinha AK (2011). The appearance of dermcidin isoform 2, a novel platelet aggregating agent in the circulation in acute myocardial infarction that inhibits insulin synthesis and the restoration by acetyl salicylic acid of its effects. *Journal of Thrombosis and Thrombolysis*.

[7] Ghosh R, Maji UK, Bhattacharya R, Sinha AK (2012). The role of dermcidin isoform 2, a two faceted atherosclerotic risk factor for coronary artery disease and the effect of acetyl salicylic acid on it. *Thrombosis*.

[8] Ghosh R, Jana P, Sinha AK (2012). The control of hyperglycemia in alloxan treated diabetic mice through the stimulation of hepatic insulin synthesis due to the production of nitric oxide. *Experimental and Clinical Endocrinology and Diabetes*.

[9] de Boer IH, Kestenbaum B, Rue TC (2008). Diabetes Control and Complications Trial (DCCT)/Epidemiology of Diabetes Interventions and Complications (EDIC) Study Research Group Insulin therapy, hyperglycemia, and hypertension in type 1 diabetes mellitus. *Archives of Internal Medicine*.

[10] Sowers JR, Standley PR, Ram JL, Jacober S, Simpson L, Rose K (1993). Hyperinsulinemia, insulin resistance, and hyperglycemia: contributing factors in the pathogenesis of hypertension and atherosclerosis. *The American Journal of Hypertension*.

[11] Chakraborty S, Khan GA, Karmohapatra SK, Bhattacharya R, Bhattacharya G, Sinha AK (2009). Purification and mechanism of action of “cortexin”, a novel antihypertensive protein hormone from kidney and its role in essential hypertension in men. *Journal of the American Society of Hypertension*.

[12] Furman MI, Benoit SE, Barnard MR (1998). Increased platelet reactivity and circulating monocyte-platelet aggregates in patients with stable coronary artery disease. *Journal of the American College of Cardiology*.

[13] Fuster V, Badimon J, Chesebro JH, Fallon JT (1996). Plaque rupture, thrombosis, and therapeutic implications. *Haemostasis*.

[14] Chakraborty K, Sinha AK (2004). The role of insulin as an antithrombotic humoral factor. *BioEssays*.

[15] Sinha AK, Bhattacharya S, Acharya K, Mazumder S (1999). Stimulation of nitric oxide synthesis and protective role of insulin in acute thrombosis in vivo. *Life Sciences*.

[16] Chakraborty K, Khan GA, Banerjee P, Ray U, Sinha AK (2003). Inhibition of human blood platelet aggregation and the stimulation of nitric oxide synthesis by aspirin. *Platelets*.

[17] Bhattacharyya M, Karmohapatra SK, Bhattacharya G, Bhattacharya R, Sinha AK (2009). The role of leucocytes in the acetyl salicylic acid (aspirin) induced nitric oxide synthesis in the production of interferon-*α*, a potent inhibitor of platelet aggregation and a thrombolytic agent. *Journal of Thrombosis and Thrombolysis*.

[18] Matsudaira P (1987). Sequence from picomole quantities of proteins electroblotted onto polyvinylidene difluoride membranes. *Journal of Biological Chemistry*.

[19] Karmohapatra SK, Chakraborty K, Kahn NN, Sinha AK (2007). The role of nitric oxide in aspirin induced thrombolysis in vitro and the purification of aspirin activated nitric oxide synthase from human blood platelets. *The American Journal of Hematology*.

[20] Knip M, Åkerblom HK (1999). Environmental factors in the pathogenesis of type 1 diabetes mellitus. *Experimental and Clinical Endocrinology and Diabetes*.

[21] Yanai H, Tomono Y, Ito K, Furutani N, Yoshida H, Tada N (2008). The underlying mechanisms for development of hypertension in the metabolic syndrome. *Nutrition Journal*.

[22] Surwit RS, Schneider MS, Feinglos MN (1992). Stress and diabetes mellitus. *Diabetes Care*.

[23] Ghosh R, Bhattacharya R, Bhattacharya G, Sinha AK (2012). The control of stress induced type I diabetes mellitus in humans through the hepatic synthesis of insulin by the stimulation of nitric oxide production. *International Journal of Biomedical Science*.

[24] Kahn NN, Acharya K, Bhattacharya S (2000). Nitric oxide: the ’second messenger’ of insulin. *IUBMB Life*.

[25] Wilcox G (2005). Insulin and insulin resistance. *Clinical Biochemist Reviews*.

[26] Bank S, Ghosh R, Jana P, Bhattacharya S, Sinha AK The diagnosis of high altitude illness by the determination of plasma dermcidin isoform 2 levels by enzyme linked immunosorbent assay.

[27] Ghosh R, Karmohapatra SK, Bhattacharya G, Kumar Sinha A (2010). The glucose-induced synthesis of insulin in liver. *Endocrine*.

[28] Ghosh R, Bhattacharyya M, Khan G (20132013). Diagnosis of essential hypertension in humans by the determination of plasma renal cortexin using enzyme-linked immunosorbent assay.

[29] Maji UK, Ghosh R, Mazumder S, Bhattacharya R, Bhattacharyya M, Sinha AK, Demir DM (2011). Control of elevated blood pressures by acetyl salicylic acid (Aspirin) in randomized patients diagnosed to be hypertensive at presentation. *Aspirin: Therapeutic Uses, Adverse Effects and Pharmacokinetics*.

[30] Sowers JR, Epstein M, Frohlich ED (2001). Diabetes, hypertension, and cardiovascular disease an update. *Hypertension*.

[31] Hennekens CH, Dyken ML, Fuster V (1997). Aspirin as a therapeutic agent in cardiovascular disease: a statement for healthcare professionals from the American heart association. *Circulation*.

[32] Yoon J-W, Jun H-S (2005). Autoimmune destruction of pancreatic *β* cells. *The American Journal of Therapeutics*.

